# Trends in the prevalence and intensity of soil-transmitted helminth (STH) infection in Ethiopia 2000 to 2023: a systematic review

**DOI:** 10.1186/s13071-025-06928-3

**Published:** 2025-08-09

**Authors:** Birhan Mengistu, Rosie Maddren, Ben Collyer, Roy M. Anderson

**Affiliations:** School of Public Health Building, Imperial College London, 90 Wood Lane, London, W12 0BZ UK

**Keywords:** STH, *A. lumbricoides*, *T. trichiura*, Hookworm, Prevalence, Intensity

## Abstract

**Background:**

Soil-transmitted helminths (STH) are a source of parasitic infections common in Ethiopia and cause stunting of growth and neurodevelopment. The aim of this review was to examine the trends in STH prevalence and intensity in Ethiopia by year, age group, and region over a period of more than two decades.

**Methods:**

A comprehensive literature review using predefined terms was conducted in PubMed, Scopus, and Web of Science. The relevant studies were screened and reviewed, and the data were extracted and recorded in an Excel spreadsheet. A random-effects model was employed to determine the pooled prevalence. Prevalence estimates and their standard errors were extracted for each period, and pairwise comparisons of estimates between consecutive periods were performed, with *P*-values computed to assess the statistical significance in the changes recorded.

**Results:**

A total of 310 studies published from 2000 to 2023, focused on STHs in Ethiopia, were included in the analysis. Of these, 298 focused on *Ascaris lumbricoides*, 250 on *Trichuris trichiura*, and 278 on hookworms. The majority of studies were conducted in the Amhara region (43.5%), followed by Oromia (26.1%). The overall prevalence of *A. lumbricoides* decreased from 13.8% (95% confidence interval [CI] 11.5%, 16.8%) before 2015 to 9.4% (95% CI 6.8%, 13.1%) after 2020, with a notable change observed between 2015 and 2019. In contrast, the prevalence of *T. trichiura* and hookworms did not show a significant change.

**Conclusions:**

Progress has been made in reducing the prevalence and intensity of *A. lumbricoides*, but there is still some way to go, which will require higher mass drug administration (MDA) coverage levels plus treatment of the whole community including adults, alongside water, sanitation, and hygiene (WaSH) interventions to prevent persistent reinfection.

**Graphical Abstract:**

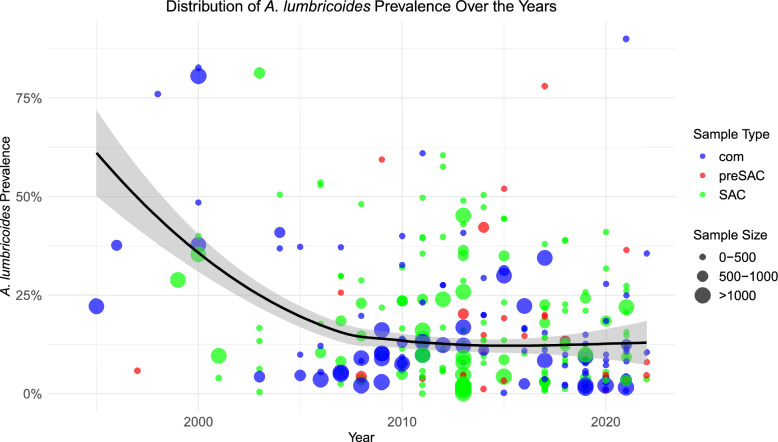

**Supplementary Information:**

The online version contains supplementary material available at 10.1186/s13071-025-06928-3.

## Background

Soil-transmitted helminth (STH) infections are caused by a group of four parasitic nematodes, namely, *Ascaris lumbricoides*, *Trichuris trichiura*, and two hookworm species (*Ancylostoma duodenale* and *Necator americanus*), along with *Strongyloides stercoralis* [1]. The parasites affect approximately one-fourth of the world’s population, mostly in sub-Saharan Africa, China, South America, and Asia, as these tropical climates are suitable for transmission. STHs are widespread in communities with limited access to sanitation and safe water supply [2]. The greatest burden of *A. lumbricoides* and *T. trichiura* is among preschool-age (pre-SAC) and school-age children (SAC), leading to growth and neurodevelopmental disorders in those heavily infected [3]. Heavy hookworm burden is more common in adults than children [4].

To control STH infections in endemic regions, the World Health Organization (WHO) recommends preventive chemotherapy, using albendazole or mebendazole, through mass drug administration (MDA) in high-risk age groups, including young children (12–23 months), pre-SAC (24–59 months), SAC (5–14 years), women of reproductive age, and pregnant mothers in their second or third trimester. Drug treatment is suggested for a group when the prevalence of STHs is equal to or greater than 20% in that group [2]. This treatment ultimately aims to decrease the prevalence of moderate and high-intensity STH infections in SAC to less than 2% [2].

Analyses based on mathematical models of transmission and control impact suggest that STH transmission can be interrupted through MDA alone, if there is high treatment coverage (80–90%) sustained over multiple rounds in endemic communities. In the longer term, however, there is a risk that transmission could return to the pre-treatment level in the absence of improvements in water, sanitation, and hygiene (WaSH) infrastructure, if or when MDA ceases [5]. Models clearly demonstrate that interruption of STH transmission is possible in low infection settings, even with moderate MDA coverage (for example, 60% in a biannual programme and 85% in an annual programme [6]). The details in such calculations depend on the intrinsic transmission potential in a specific area as defined by the magnitude of the basic reproductive number, R_0_.

There are notable gains in reducing STH burden through MDA in terms of infection exposure, but reinfection rates remain high if there is insufficient MDA coverage across all age groups to eliminate pockets of infection that continually seed transmission [7]. Ideally, MDA should be targeted at all age groups to eliminate these pockets of infection at high coverage, and in parallel, access to adequate WaSH services will be crucial in the longer term to facilitate STH transmission interruption. The main limitations to effective STH control in regions of endemic infection include low MDA coverage as a percentage of the target group, the use of diagnostic methods with low sensitivity, and groups of people who continually miss repeated rounds of MDA (i.e., low compliance) [8].

STHs continue to be a significant public health problem in Ethiopia, with varying prevalence rates reported across different surveys. A 10-year analysis of published data revealed that *A. lumbricoides*, *T. trichiura*, and hookworms are the predominant cause of infections, with prevalence of 17.6%, 12.4%, and 7.2%, respectively. The highest prevalence was found in the Southern Nations, Nationalities, and Peoples’ Region (SNNPR) (now divided into three regions, and referred to as the Southern region in this review), followed by the Oromia region [9].

A national mapping programme conducted in 2015 reported an overall STH prevalence of 21.7%, and individually 12.8% for *A. lumbricoides*, 7.6% for hookworm infection (both species combined), and 5.9% for *T. trichiura*. The prevalence of *S. stercoralis* was not recorded in this survey, but this parasite is known to be prevalent in some areas of Ethiopia [10]. Similarly, the national school health and nutrition survey conducted between 2005 and 2006 revealed a prevalence of 20.7% for *A. lumbricoides*, 6% for *T. trichiura*, and 7.4% for hookworms among school children [11].

The national deworming programme was implemented with a focus on SAC, alongside remapping activities conducted in 2020. However, analyses of trends in STH infections in Ethiopia and the impact of existing control programmes remain few and far between. This review aims to provide such a trend analysis of changes over time in the prevalence (and where possible the intensity of infection) of STHs excluding *S. stercoralis* by year, age group, and region of Ethiopia where samples were taken for parasitological analysis. *Strongyloides stercoralis* was excluded as it was not consistently reported across different studies and was not targeted in the STH control programme.

## Methods

### Data and search strategy

Studies published from 2000 to 2023 were reviewed, focusing on those in the English language. The search was performed in PubMed, Scopus, and Web of Science. The following search terms were used: “soil-transmitted helminths” OR “nematode” OR “*Necator*” OR “*Ancylostoma*” OR “*Ascaris*” OR “*Trichuris*” OR “hookworm” OR “roundworm,” OR “Geo-helminths” OR “whipworm”.

### Inclusion criteria

The studies identified by the search terms were downloaded into Mendeley and saved after removing duplicates. First, they were screened based on their abstracts, and then the whole article was reviewed to determine the content for inclusion in the review-based analysis. The publications included in this review reported prevalence (and where possible, intensity), sample size, sample type, and the diagnostic methods employed in survey studies.

### Exclusion criteria

Publications were excluded from the review if they failed to meet the inclusion criteria or did not explicitly report the prevalence. Publications classified as systematic reviews and studies that utilized a purposive sampling method were also excluded.

### Data extraction and analysis

The following data were extracted from the publications: author names, year of publication, year of study, region, prevalence and intensity of STH species, drugs used, study type, sample size, diagnostic tests used, and latitude and longitude of study areas. The heterogeneity among the studies was evaluated using the *I*^2^ statistic, with a value of 75% or above indicating significant variation among the studies. Anticipating a high level of heterogeneity, the studies were further stratified by period (before 2015, between 2015 and 2019, and after 2020), region in Ethiopia, and sample population type. A random-effects model was employed to determine the pooled prevalence. Prevalence estimates and their standard error were extracted for each period, and pairwise comparisons of estimates between consecutive periods were performed, with *P*-values computed to assess the statistical significance in the changes recorded. Relative changes in prevalence were also calculated between periods. Mann–Whitney *U* tests were used to compare the weighted mean egg counts by the periods. The maps for each species were created using the latitudes and longitudes of the study coordinates. A non-linear model was fitted to describe the relationship between prevalence and intensity, as well as to estimate k values through maximum likelihood, which inversely reflect the degree of parasite aggregation within the human host population.

## Results

**Fig. 1:**
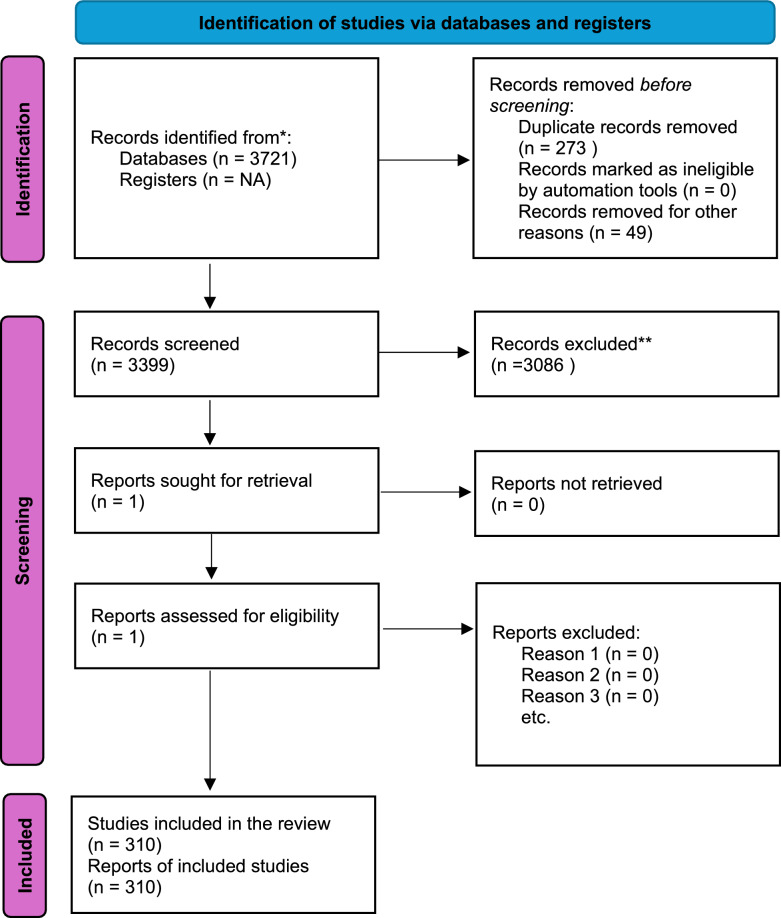
Diagram illustrating the systematic review process

A total of 3721 studies focused on STHs in Ethiopia and published from 2000 to 2023 were identified; 341 were excluded, leaving 310 included for analysis (Fig. 1). Out of these, 78.7% were cross-sectional studies involving a total of 429,022 participants. A total of 172 (55.5%) studies focused on SAC between 5 and 14 years of age, while 113 (36.5%) and 25 (8.0%) examined the whole community and pre-SAC (ages 4 years and younger), respectively. The diagnostic methods employed varied, with 126 studies utilizing the Kato-Katz (KK) technique alone or in combination with other tests, 61 employing direct microscopy, 45 using the formalin–ether concentration technique (FECT), and others applying different techniques including quantitative polymerase chain reaction (qPCR), agar plate culture, and centrifugation methods.

Among the studies, 298 reported the prevalence of *A. lumbricoides*, but only 61 (20.5%) reported data on infection intensity. For *T. trichiura*, 250 studies reported prevalence, while only 57 (22.0%) provided intensity data. Similarly, 278 studies reported the prevalence of hookworm, but just 56 (20.1%) included intensity figures. Regionally, 155 (43.5%) surveys were conducted in the Amhara region, 81 (26.1%) in Oromia, and 64 (20.6%) in the Southern region, with the remaining studies scattered across the other regions. Notably, the studies excluded participants who had been treated with antiparasitic medication 2 weeks to 6 months before the survey, ensuring the accuracy of the findings assigned to a given year.

### Trends in *A. lumbricoides* prevalence and intensity

**Fig. 2:**
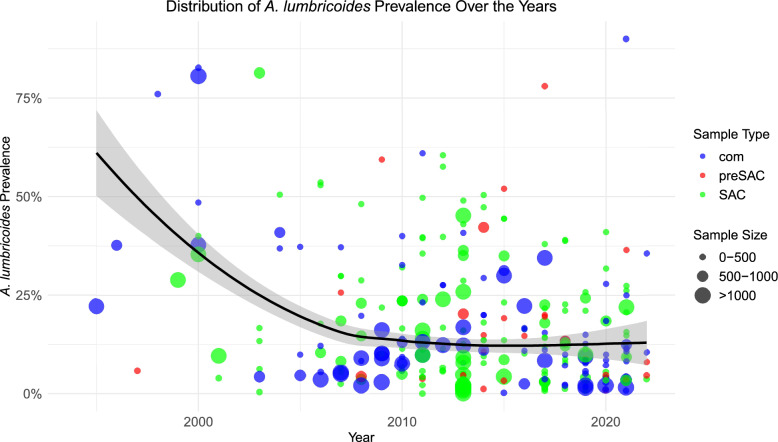
Trends in *A. lumbricoides* prevalence over the years in Ethiopia. *Note*: “com” includes studies involving all age groups not defined as pre-SAC or SAC (“pre-SAC” = children aged less than 5 years, “SAC” = children aged 5 to 14 years)

In Ethiopia, the prevalence of *A. lumbricoides* decreased from an overall prevalence of 13.8% (95% confidence interval [CI] 11.5%, 16.8%, *n* = 160) before 2015 to 9.5% (7.5%, 11.9%, *n* = 87) between 2015 and 2019, and a further decrease was seen after 2020 to a prevalence of 9.4% (CI 6.8%, 13.1%, *n* = 48) (Fig. 2). Regionally, the prevalence decreased significantly in Amhara, with a notable change of 54.8% between 2015 and 2019 (*P* = 0.01). In Tigray and Oromia, non-significant reductions of 42.5% and 12.9% were observed between 2015 and 2019, followed by 13.9% and 25.4% after 2020, while in the Southern region, the prevalence increased slightly but non-significantly after 2020.

The greatest reduction was observed in samples taken from the community as a whole, which decreased from 20.0% before 2015 to 8.1% between 2015 and 2019 (*P*<0.0001).

The prevalence decreased non-significantly among SAC from 14.6% before 2015 to 10.6% between 2015 and 2019 (*P* = 0.1), and it increased to 13.1% after 2020, also non-significantly (*P* = 0.4). The prevalence among pre-SAC increased during 2015 and 2019 but saw a notable reduction after 2020 (*P* = 0.04). The mean *A. lumbricoides* egg count showed a non-significant increase from 2015 to 2019 but decreased slightly after 2020 (*P* = 0.9) (Figure S1).

**Fig. 3:**
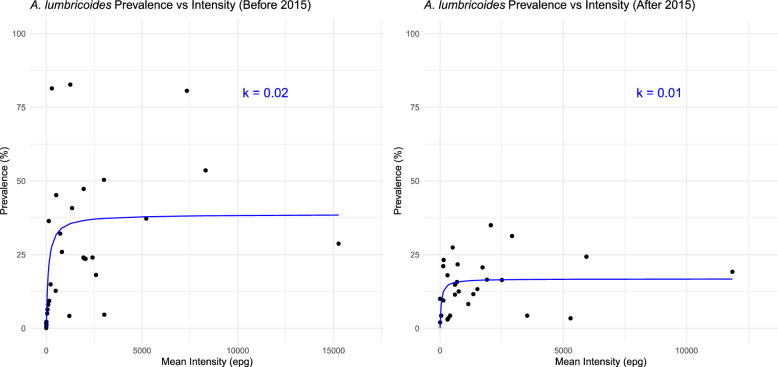
*Ascaris lumbricoides* prevalence and aggregation over the period. **A** The relationship between the prevalence and intensity of *A. lumbricoides*, where the solid line is the best-fit negative binomial relationship between prevalence using prevalence and intensity to estimate k. Prevalence (P) is recorded as a percentage and mean intensity is recorded as M, as assessed by eggs per gram (epg) in faeces—where P = 100 [1 − (1 + M/k) − k] by value [12]. **B** The relationship between the prevalence and intensity of *A. lumbricoides* observed by the line through the data after 2015, with the k value

The relationship between *A. lumbricoides* prevalence and intensity, as observed in Fig. 3, shows that prevalence increased rapidly over low mean intensity levels as they increased and then reached a plateau level where its magnitude is defined by the parasite aggregation parameter k. The degree of aggregation measured inversely using the negative binomial parameter k (low value indicates lower prevalence and higher aggregation) was 0.02 in the period before 2015 and decreased to 0.01 afterward, indicating increased levels of aggregation and concomitantly diminishing responsiveness of prevalence to changes in intensity over time.

### Trends in *T. trichiura* prevalence and intensity

**Fig. 4:**
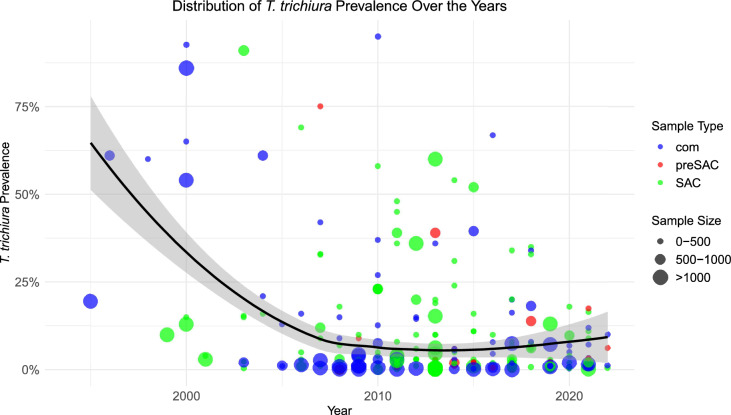
Trends in *T. trichiura* prevalence over the years in Ethiopia

*Trichuris trichiura* prevalence showed a non-significant declining trend from 5.1% (3.7%, 7.1%, *n* = 151) before 2015 to 3.6% (2.4%, 5.4%, *n* = 63) between 2015 and 2019, and further declined non-significantly after 2020 to 3.0% (2.0%, 4.5%, *n* = 75) (Fig. 4). The prevalence reduction by region was highest in the Southern region, which saw a 68.1% decrease (*P* = 0.03) between 2015 and 2019, though it increased non-significantly after 2020 (*P* = 0.1). In Amhara, the prevalence consistently decreased across the two periods, with a significant reduction observed between 2015 and 2019 (*P* = 0.01) and after 2020 (*P* = 0.2). In contrast, Tigray experienced a non-significant reduction in prevalence. Similarly, in Oromia, a non-significant increase (*P* = 0.8) was found between 2015 and 2019.

The weighted mean *T. trichiura* egg count increased non-significantly from 47.0 (20.2, 161.6) before 2015 to 98.3 (39.7, 269.9) between 2015 and 2019, to 435.2 (109.1, 906.2) after 2020 (Figure S2).

**Fig. 5:**
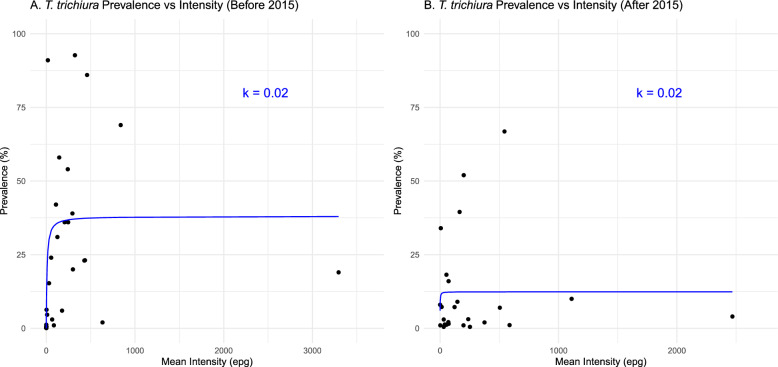
Prevalence and aggregation of *T. trichiura* over time. **A** The relationship between the prevalence and intensity of *T. trichiura* observed by the line through the data before 2015 with the k value. **B** The relationship between the prevalence and intensity of *T. trichiura* observed by the line through the data after 2015 with the k value. *epg* eggs per gram

The degree of aggregation for *T. trichiura*, calculated using k, remained unchanged between the periods before 2015 and after 2015 (Fig. 5), suggesting little change in the dynamics of this infection.

### Trends in hookworm prevalence and intensity

**Fig. 6:**
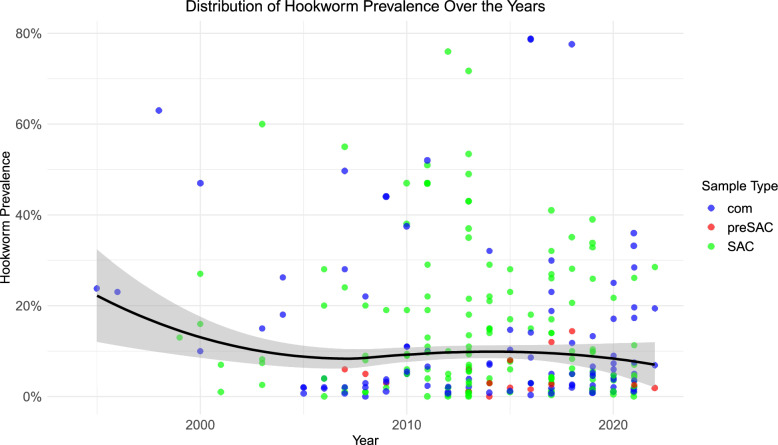
Trends in hookworm prevalence over the years in Ethiopia

Hookworm prevalence, unlike that for the other species, showed a non-significant decrease from 7.9% (6.3%, 10.1%, *n* = 144) before 2015 to 6.8% (5.1%, 9.0%, *n* = 90) between 2015 and 2019, followed by a slight decline to 5.8% (4.0%, 8.2%, *n* = 42) after 2020 (*P* = 0.4) (Fig. 6). The Amhara region showed the highest reduction (by 42.9%), dropping from 16.3% before 2015 to 9.3% between 2015 and 2019 (*P* = 0.05%), though it increased slightly after 2020 (*P* = 0.06). There was a non-significant decrease in the Southern region and Oromia across the periods. In contrast, the Tigray region saw a non-significant increase between 2015 and 2019, followed by a non-significant decline after 2020.

The weighted mean hookworm egg count increased non-significantly from 31.1 (22.3, 54.6) before 2015 to 141.1 (51.7, 387.4) between 2015 and 2019, to 418.0 (176.5, 795.8) (Figure S3).

**Fig. 7:**
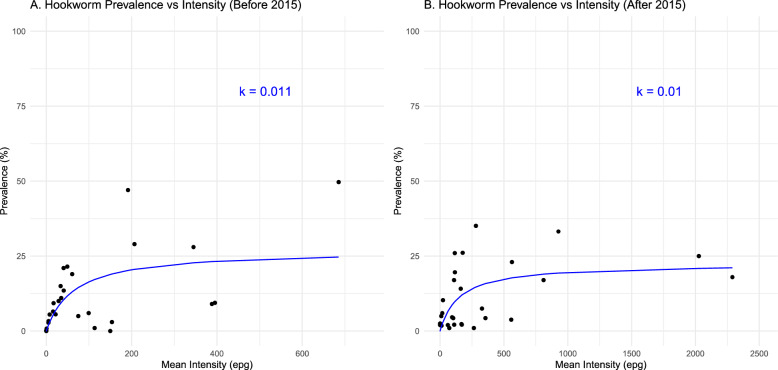
Hookworm prevalence and aggregation over time. **A** The relationship between the prevalence and intensity of hookworm observed by the line through the data before 2015 with the k value. **B** The relationship between the prevalence and intensity of hookworm observed by the line through the data after 2015, with the k value. *epg* eggs per gram

The k value for hookworm changed slightly between the periods before 2015 and after 2015, decreasing from 0.011 to 0.1 (Fig. 7).

### STH MDA treatment coverage

**Fig. 8:**
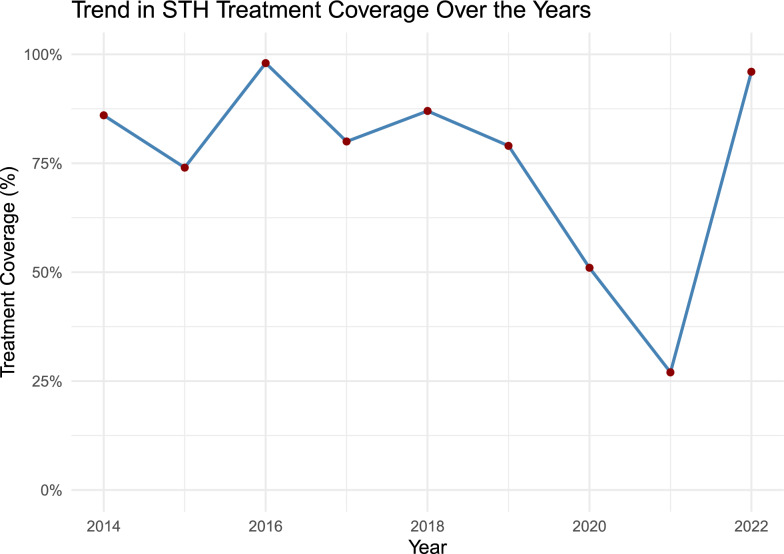
Treatment coverage in Ethiopia over the years. Source: ESPEN: Ethiopia. Expanded Special Project for Elimination of Neglected Tropical Diseases. Data reported by the Ethiopian Ministry of Health to WHO. Additional file 2 Figure S1: Trends in *A. lumbricoides* mean egg count over the years (intensity of infection) (Box: describes the interquartile range [IQR] 25–75%). Whiskers: 1.5 times the IQR. Red dots: outliers)

Based on the Expanded Special Project for Elimination of Neglected Tropical Diseases (ESPEN) data, the reported treatment coverage for STHs recorded by the Ministry of Health remained above 75% until 2019 but decreased considerably in 2020 and 2021 during the coronavirus disease 2019 (COVID-19) pandemic, before returning to high coverage of 96% in 2022 (Fig. 8). No treatment data are available for pre-SAC and adults, since the existing control programme only targets SAC.

### Demography in Ethiopia

The population of Ethiopia has been growing rapidly at a rate of 2.5% per annum in recent years, increasing from 100 million in 2017 to 127 million in 2023 [12]. According to the US Census Bureau, nearly 40% of the population is under the age of 15 years, which puts significant pressure on drug supply and delivery resources [13].

## Discussion

This systematic review reports on the progress in the control of STH infections, specifically those caused by *A. lumbricoides*, *T. trichiura*, and hookworm, from 2000 to 2023. The study reports on data extracted from 310 papers, which provided quantitative information on the burden of STH infection stratified by year, region, and age group. A significant proportion of the studies focused on SAC, reflecting the current WHO guidelines for treatment and monitoring plus evaluation.

Several diagnostics were used, including direct microscopy and FECT among others, despite WHO recommendations for the use of KK as the standard for monitoring and evaluation (M&E) of STH infections [14, 15]. The use of varied techniques can result in differences in the measurement of prevalence and intensity. Furthermore, only a small proportion of studies looked at the intensity of infection. Variability in age classification across studies also affected comparisons. Additionally, non-validated treatment reports may lead to an overestimation of MDA implementation coverage.

STH M&E programmes need to address both the inconsistencies in age group classification and standardization of diagnostic methods, particularly in low-prevalence settings. WHO recommends the KK method as choice of diagnostic and survey in SAC as a proxy indicator, without disaggregating data by age or sex [16]. An effective M&E programme should be comprehensive (including prevalence, intensity, demographic, and treatment data), focusing on robust data collection (based on adequate sample size, quality control process, and highly sensitive and specific diagnostic techniques), cost-effectiveness (determining the best value for resources spent), and community involvement. By standardizing M&E protocols and investing in capacity-building, a significant impact could be made in the fight against STHs and improving health outcomes in Ethiopia over the coming decade.

The overall *A. lumbricoides* prevalence dropped from 13.8% to 9.4% (Table S1) over the last decade, indicating the effectiveness of the SAC-based deworming policy, which was launched in 2001 [17], and improvements in WaSH services in the country. Deworming projects were carried out in many parts of Ethiopia over the study period, which were enhanced by the launch of a national deworming programme in 2015, enabling the distribution of an estimated 92.7 million treatments for STHs [18]. Prevalence was substantially reduced in the Amhara region, which experienced a decrease of 54.8% between 2015 and 2019. This reduction can potentially be attributed to high and sustained MDA coverage and improved programmatic follow-up. However, a mixed picture emerged in other regions like Tigray and Oromia, which showed non-significant reductions. This may be due to differences in programme commitment, baseline endemicity, and population movement that may have impacted these regional patterns. Additionally, an increasing trend was noted in the Southern region, despite relatively improved reported stability. These factors may highlight challenges specific to implementation quality, coverage consistency, or data reporting in that region, underscoring the need for more targeted investigations into programme gaps and regional disparities.

Community-based estimates of the prevalence of infection showed a significant reduction for *A. lumbricoides*, which declined from 20.0% before 2015 to 8.1% between 2015 and 2019. This reflects the benefit of deworming and WaSH activities in other groups not targeted by MDA, as the overall worm burden decreased, thereby reducing the risk of exposure [19] (the indirect benefits to untreated groups generated by treatment in other population groupings). Despite this, the prevalence among SAC showed a non-significant increase after 2020, suggesting that further efforts are required to increase MDA coverage and improve the integration of treatment with other interventions such as WaSH improvements to further reduce transmission and sustain the results achieved by MDA. The prevalence among pre-SAC increased between 2015 and 2019, but decreased after 2020. This highlights the need for treating this age group in combination with SAC.

The intensity of *A. lumbricoides* infection did not show a significant change despite the reduction in prevalence. This could be due to the aggregation of parasites in certain groups, and with a few individuals harbouring a higher number of eggs, as the prevalence decreases, especially in untreated adults. As prevalence decreases, the aggregation of worms within the human population increases, leading to hotspots of infection in a few individuals [20]. This indicates the importance of both identifying individuals with high numbers of eggs and treating adult age groups to enhance control impact.

The impact of deworming interventions was not evident in the prevalence of *T. trichiura*, which exhibited a non-significant reduction from 5.1% before 2015 to 3.0% after 2020, as indicated by the high k value. This suggests the limitation of MDA interventions for this parasite, given the low efficacy of albendazole and mebendazole for this STH species. Several studies have reported lower efficacy of albendazole and mebendazole for *T. trichiura* [21–24].

This finding was in line with a recent meta-analysis study, which found minimal reduction of *T. trichiura* among children and adolescents [25, 26]. However, one systematic review conducted in Ethiopia [27] reported a significant reduction in this species. A small reduction in *T. trichiura* prevalence in this review suggests the need to incorporate WaSH services to further decrease the transmission and also to consider what drug combinations might effectively treat *T. trichiura* infection [28].

The reduction in prevalence for *T. trichiura* was greatest in the Southern region between 2015 and 2019, but an increase was observed after 2020, potentially related to missed MDA rounds due to national remapping activities and the COVID-19 pandemic, which redirected the focus and resources of the Ethiopian health system.

A consistent reduction in *T. trichiura* prevalence was found in the Amhara region, but changes in Oromia and Tigray were not significant. This highlights regional differences in the implementation of the MDA programme, potentially attributed to factors such as proper planning, logistic management, conflict, and the rapidly growing population that influences drug demand.

There was a non-significant reduction in the prevalence of *T. trichiura* in both the SAC and the community groups, though the prevalence in the community increased after 2020. No changes were seen in pre-SAC during either period. This highlights that exposure outside of SAC is common and supports the need to reconsider control strategies. Additionally, a study conducted in Indonesia reported a high prevalence of *T. trichiura* infection among pre-SAC [29].

The intensity of *T. trichiura* infections varies over time, with a gradual and non-significant increase observed across the periods. This indicates ongoing transmission and increasing morbidity caused by the worms (Table S1).

The overall rate of hookworm infection saw a non-significant reduction. This may be influenced by several factors, including the limited efficacy of albendazole or mebendazole compared to *A. lumbricoides* [30, 31]. Additionally, even with ongoing MDA, transmission could be facilitated by the ability of hookworm larvae to stay in the environment for longer periods [32]. Furthermore, variation is found in the epidemiology and complexity of helminth infections, with hookworm infection peaks and plateaus in the adult age groups [33]. As such, MDA targeting SAC is less likely to make a significant impact on this parasite [34]. This, in general, highlights the need for integration of interventions with WaSH improvements.

Regionally, the prevalence of hookworm showed a substantial reduction in the Amhara region between 2015 and 2019, with a decrease from 16.3% to 9.9%. The other regions had mixed outcomes, with no significant changes recorded.

The variation in hookworm prevalence by age group, where SAC saw decreases but an increasing trend was seen in the general community, highlights the need to involve adults in MDA treatment. This agrees with a systematic review from the Philippines, which reports a limited effect of chemotherapy among adults [34].

The mean hookworm egg count increased steadily across the various periods, attributable to active transmission and ineffective deworming efforts targeting SAC (Table S1). This is in line with several studies reporting that the transmission dynamics of hookworm were higher among adults [35–37]. Hence, extending deworming to include this group increases the effectiveness of control strategies.

The findings from the systematic review suggest that M&E activities should target all age groups, not just SAC, with enhanced MDA treatment coverage data, which is verified by coverage surveys. In addition, progress has been made in reducing the prevalence and intensity of STHs, but a comprehensive intervention is crucial to sustaining gains. This includes higher MDA coverage levels, expanding treatment to the whole community, including adults, but also improvement in WaSH services to address reinfection.

There were a limited number of published papers documenting STH infection levels in Gambella, Somali, Afar, Benishangul, Dire Dawa, Addis Abeba, and Harari, hindering comprehensive comparisons of trends in other regions of Ethiopia. Additionally, the significant heterogeneity regarding methodology, population groups, diagnostic methods, sampling strategies, and classifications of geographical settings may impact the comparisons of data gathered from different studies, despite employing random-effects analysis to account for variability. Furthermore, data on the intensity of infection, as opposed to prevalence, were scarce.

## Conclusions

The prevalence of *A. lumbricoides* infections showed a significant reduction over the study periods, but changes in infection levels for *T. trichiura* and hookworm were much less. This is probably due to several factors, including low drug efficacy in treating *T. trichiura* and not treating adults for hookworm infection, given that this demographic group harbours higher burden than children. Despite the reduction in *A. lumbricoides* prevalence, the mean egg counts showed a non-significant change over time, suggesting that infection may be aggregated in a few individuals, since aggregation levels rise as MDA reduces worm burden [20]. Integrating MDA programmes with WaSH efforts is crucial to the long-term control of STH transmission and to sustaining reductions in infection levels.

## Supplementary Information

Additional file 1. Table S1. Prevalence and intensity of infection by species

Additional file 2. Figure S1. Trends in *A. lumbricoides* mean egg count over the years. A. lumbricoides mean egg count over the years (intensity of infection) (Box: describes the interquartile range [IQR] 25–75%). Whiskers: 1.5 times the IQR. Red dots: outliers) View

Additional file 3. Figure S2. Trends in *T. trichiura* mean egg count over the years

Additional file 4. Figure S3. Trends in hookworm mean egg count over the years

## Data Availability

Data supporting the main conclusions of this study are included in the manuscript.

## Abbreviations

CI: Confidence interval
COVID: Coronavirus disease
FECT: Formalin–ether concentration technique
EPG: Egg per gram ESPEN: Expanded Special Project for Elimination of Neglected Tropical Diseases
IQR: Interquartile range
KK: Kato-Katz
M&E: Monitoring and evaluation
MDA: Mass drug administration NTDS: Neglected Tropical Disease
Pre-SAC: Preschool-age children
qPCR: Quantitative polymerase chain reaction
SAC: School-age children
SNNP: Southern Nations, Nationalities, and Peoples
STH: Soil-transmitted helminth
WaSH: Water, sanitation, and hygiene
WHO: World Health Organization

## References

[1] Centers for Disease Control and Prevention. CDC - Soil-Transmitted Helminths. 2022

[2] World Health Organization. Preventive Chemotherapy to Control Soil-transmitted Helminth Infections in At-risk Populations. Geneva: World Health Organization; 2017. 29578660

[3] Kretchy J-P. Soil-Transmissible Helminths Infections; diagnosis, transmission dynamics, and disease management strategies in low-and middle-income countries [Internet]. IntechOpen; 2024. 10.5772/intechopen.102829

[4] Hotez PJ, Bethony J, Bottazzi ME, Brooker S, Buss P. Hookworm: “The Great Infection of Mankind.” PLOS Med. 2005;2:e67. 10.1371/journal.pmed.0020067. 15783256 10.1371/journal.pmed.0020067 PMC1069663

[5] Anderson R, Truscott J, Hollingsworth TD. The coverage and frequency of mass drug administration required to eliminate persistent transmission of soil-transmitted helminths. Philos Trans R Soc B Biol Sci. 2014;369:1645. 10.1098/rstb.2013.0435 PMC4024228 24821921

[6] Chong NS, Smith SR, Werkman M, Anderson RM. Modelling the ability of mass drug administration to interrupt soil-transmitted helminth transmission: community-based deworming in Kenya as a case study. PLoS Negl Trop Dis. 2021;15:8. 10.1371/journal.pntd.0009625 PMC8360579 34339450

[7] Ásbjörnsdóttir KH, Means AR, Werkman M, Walson JL. Prospects for elimination of soil-transmitted helminths. Curr Opin Infect Dis. 2017;30:482–8. 28700363 10.1097/QCO.0000000000000395 PMC7680933

[8] Becker SL, Liwanag HJ, Snyder JS, Akogun O, Belizario V, Freeman MC, et al. Toward the 2020 goal of soil-transmitted helminthiasis control and elimination. PLoS Negl Trop Dis. 2018;12:e0006606. 10.1371/journal.pntd.0006606. 30106975 10.1371/journal.pntd.0006606 PMC6091919

[9] Aemiro A, Menkir S, Tegen D, Tola G. Prevalence of soil-transmitted helminthes and associated risk factors among people of Ethiopia: a systematic review and meta-analysis. Infect Dis (Auckl). 2022;15:11786337211055436. 10.1177/11786337211055437 PMC8958720 35356097

[10] Hailu T, Nibret E, Amor A, Munshea A. *Strongyloides stercoralis* infection in Ethiopia: systematic review and meta-analysis on prevalence and diagnostic methods. Helminthol. 2021;58:17–27. 10.2478/helm-2021-0010 PMC7912231 33664615

[11] Hall A, Kassa T, Demissie T, Degefie T, Lee S. National survey of the health and nutrition of schoolchildren in Ethiopia. Trop Med Int Heal. 2008;13:1518–26. 10.1111/j.1365-3156.2008.02168.x. 10.1111/j.1365-3156.2008.02168.x 18983269

[12] Ethiopia | Data. https://data.worldbank.org/country/ethiopia. Accessed 4 Oct 2024

[13] Population Clock_ World [Internet]. 2016. https://www.census.gov/popclock/world/et. Accessed 27 Jun 2025

[14] World Health Organization. Preventive chemotherapy in human helminthiasis – coordinated use of anthelminthic drugs in control interventions. Geneva: World Health Organization; 2006.

[15] WHO. WHO Guideline on control and elimination of human schistosomiasis. Geneva: World Health Organization, 2022. 142 p. 35235279

[16] World Health Organization. Assessing schistosomiasis and soil-transmitted helminthiases control programmes. Monitoring and evaluation framework. 2024

[17] Montresor A, Crompton DWT, Gyorkos TW, Savioli L. Helminth control in school-age children: a guide for managers of control programmes. World Health Organization: Geneva.

[18] Ministry of Health. The Third National Neglected Tropical Diseases Strategic Plan 2021–2025. 2021.

[19] Dhakal S, Karim MJ, Al Kawsar A, Irish J, Rahman M, Tupps C, et al. Post-intervention epidemiology of STH in Bangladesh: data to sustain the gains. PLoS Negl Trop Dis. 2020;14:e0008597. 33284834 10.1371/journal.pntd.0008597 PMC7746288

[20] Mayer J, Collyer BS, Maddren R, Abtew B, Liyew EF, Chernet M, et al. Patterns of soil-transmitted helminth aggregation in the human host population after several years of intensive mass drug administration. Trans R Soc Trop Med Hyg. 2024;118:829–31. 10.1093/trstmh/trae059. 39328058 10.1093/trstmh/trae059 PMC11638107

[21] Grolimund CM, Utzinger J, Coulibaly JT, Sayasone S, Ali SM, Keiser J, et al. Modeling transmission mechanism to infer treatment efficacy of different drugs and combination therapy against *Trichuris trichiura*. Sci Rep. 2024;14:23543. 39384803 10.1038/s41598-024-73164-7 PMC11464734

[22] Reduced efficacy of single-dose albendazole against *Ascaris lumbricoides*, and *Trichuris trichiura*, and high reinfection rate after cure among school children in southern Ethiopia: a prospective cohort study. Infect Dis Poverty. 2024;13:1–11. 10.1186/s40249-024-01176-6. 38246985 10.1186/s40249-024-01176-6 PMC10802031

[23] Mohammed OM, Khatlan SADM, Noori SS, Salam I. Efficacy and safety of mebendazole in the treatment of intestinal helminth Infections. South Asian Res J Pharm Sci. 2024;6:162–8.

[24] Matamoros G, Sanchez A, Cimino R, Krolewiecki A, Mejia R. A comparison of the diagnostic capability of Kato-Katz and real-time PCR for the assessment of treatment efficacy of ivermectin and albendazole combination against *T. trichiura* infections. PLoS Negl Trop Dis. 2024;18:e0012677. 10.1371/journal.pntd.0012677. 39561184 10.1371/journal.pntd.0012677 PMC11614246

[25] Salam RA, Maredia H, Das JK, Lassi ZS, Bhutta ZA. Community-based interventions for the prevention and control of helmintic neglected tropical diseases. Infect Dis POVERTY. 2014;3:23. 25114793 10.1186/2049-9957-3-23 PMC4128617

[26] Naqvi FA, Das JK, Salam RA, Raza SF, Lassi ZS, Bhutta ZA. Interventions for neglected tropical diseases among children and adolescents: a meta-analysis. Pediatrics. 2022;149:6. 10.1542/peds.2021-053852E 35503336

[27] Maddren R, Phillips A, Ower A, Landeryou T, Mengistu B, Anjulo U, et al. Soil-transmitted helminths and schistosome infections in Ethiopia: a systematic review of progress in their control over the past 20 years. Parasites Vectors. 2021;14:1–15. 10.1186/s13071-021-04600-0. 33546757 10.1186/s13071-021-04600-0 PMC7866680

[28] Hürlimann E, Keller L, Patel C, Welsche S, Hattendorf J, Ali SM, et al. Efficacy and safety of co-administered ivermectin and albendazole in school-aged children and adults infected with *Trichuris trichiura* in Côte d’Ivoire, Laos, and Pemba Island, Tanzania: a double-blind, parallel-group, phase 3, randomised controlled trial. Lancet Infect Dis. 2022;22:123–35. 34856181 10.1016/S1473-3099(21)00421-7

[29] Djuardi Y, Lazarus G, Stefanie D, Fahmida U, Ariawan I, Supali T. Soil-transmitted helminth infection, anemia, and malnutrition among preschool-age children in Nangapanda subdistrict, Indonesia. PLoS Negl Trop Dis. 2021;15:e0009506. 34138863 10.1371/journal.pntd.0009506 PMC8253427

[30] Soukhathammavong PA, Sayasone S, Phongluxa K, Xayaseng V, Utzinger J, Vounatsou P, et al. Low Efficacy of Single-Dose Albendazole and Mebendazole against Hookworm and Effect on Concomitant Helminth Infection in Lao PDR. PLoS Negl Trop Dis. 2012;6:e1417. 10.1371/journal.pntd.0001417. 22235353 10.1371/journal.pntd.0001417 PMC3250499

[31] Adegnika AA, Zinsou JF, Issifou S, Ateba-Ngoa U, Kassa RF, Feugap EN, et al. Randomized, controlled, assessor-blind clinical trial to assess the efficacy of single- versus repeated-dose albendazole to treat *Ascaris lumbricoides*, *Trichuris trichiura*, and Hookworm Infection. Antimicrob Agents Chemother. 2014;58:2535. 24550339 10.1128/AAC.01317-13 PMC3993258

[32] Na-Ek P, Sanpool O, Jongthawin J, Anamnart W, Intapan PM, Chamavit P, et al. Restoration of hookworm egg development after prolonged storage in stool suspension. Parasitol Res. 2016;115:2817–23. 10.1007/s00436-016-5031-4. 27053130 10.1007/s00436-016-5031-4

[33] Anderson RM, May RM. Population dynamics of human helminth infections: control by chemotherapy. Nature. 1982;297:557–63. 7088139 10.1038/297557a0

[34] Witek-McManus S, Simwanza J, Chisambi AB, Kepha S, Kamwendo Z, Mbwinja A, et al. Epidemiology of soil-transmitted helminths following sustained implementation of routine preventive chemotherapy: demographics and baseline results of a cluster randomised trial in southern Malawi. PLoS Negl Trop Dis. 2021;15:e0009292. 33979325 10.1371/journal.pntd.0009292 PMC8224978

[35] Trinos JPCRD, Wulandari LPL, Clarke N, Belizario V, Kaldor J, Nery SV. Prevalence of soil-transmitted helminth infections, schistosomiasis, and lymphatic filariasis before and after preventive chemotherapy initiation in the Philippines: A systematic review and meta-analysis. PLoS Negl Trop Dis. 2021;15:e0010026. 10.1371/journal.pntd.0010026. 34928944 10.1371/journal.pntd.0010026 PMC8722724

[36] Ohiolei JA, Isaac C, Omorodion OA. A review of soil transmitted helminthiasis in Nigeria. Asian Pacific J Trop Dis. 2017;7:841–8.

[37] Avokpaho EFGA, Houngbégnon P, Accrombessi M, Atindégla E, Yard E, Means AR, et al. Factors associated with soil-transmitted helminths infection in Benin: findings from the DeWorm3 study. PLoS Negl Trop Dis. 2021;15:e0009646. 34403424 10.1371/journal.pntd.0009646 PMC8396766

