# Supplementary material for: Trends in the prevalence and intensity of soil-transmitted helminth (STH) infection in Ethiopia 2000 to 2023: a systematic review
Source: Parasit Vectors. 2025 Aug 9;18:340. doi: 10.1186/s13071-025-06928-3 (PMC12335801; doi:10.1186/s13071-025-06928-3)
Supplement: Supplementary file 1 — Additional file 1. Table S1. Prevalence and intensity of infection by species [file 13071_2025_6928_MOESM1_ESM.pdf]

Additional file 1: Table S1. Prevalence and intensity of infection by species

	Before 2015	Between 2015 to 2019	After 2020
A.Lumbricoides prevalence (95%CI)	13.9% (11.5%, 16.6%)	9.5% (7.5%, 11.9%)	9.4% (6.8%, 13.1%)
T. trichiura prevalence (95%CI)	5.1% (3.7% - 7.1%)	3.6% (2.4%, 5.4%)	3.0% (2.0%, 4.5%)
Hookworm prevalence (95%CI)	7.9% (6.3% ,10.1%)	(5.1%, 9.0%)	(4.0%, 8.2%)
A.Lumbricoides mean egg count (epg) (95%CI)	618.7 (247.4, 1917.8)	1217.8 (461.6, 3243.7)	1002.0 (578.9, 1662.3)
T. trichiura mean egg count (epg) (95%CI)	47.0(20.2, 161.6)	98.3 (39.7, 269.9)	435.2 (109.1, 906.2)
Mean hookworm egg count (epg) (95%CI)	31.1 (22.3, 54.6)	141.1 (51.7, 387.4)	418.0 (176.5, 795.8)
