# Supplementary material for: Trends in the prevalence and intensity of soil-transmitted helminth (STH) infection in Ethiopia 2000 to 2023: a systematic review
Source: Parasit Vectors. 2025 Aug 9;18:340. doi: 10.1186/s13071-025-06928-3 (PMC12335801; doi:10.1186/s13071-025-06928-3)
Supplement: Supplementary file 2 — Additional file 2. Figure S1. Trends in A. lumbricoides mean egg count over the years. A. lumbricoides mean egg count over the years (intensity of infection) (Box: describes the interquartile range [IQR] 25–75%). Whiskers: 1.5 times the IQR. Red dots: outliers) View [file 13071_2025_6928_MOESM2_ESM.pdf]

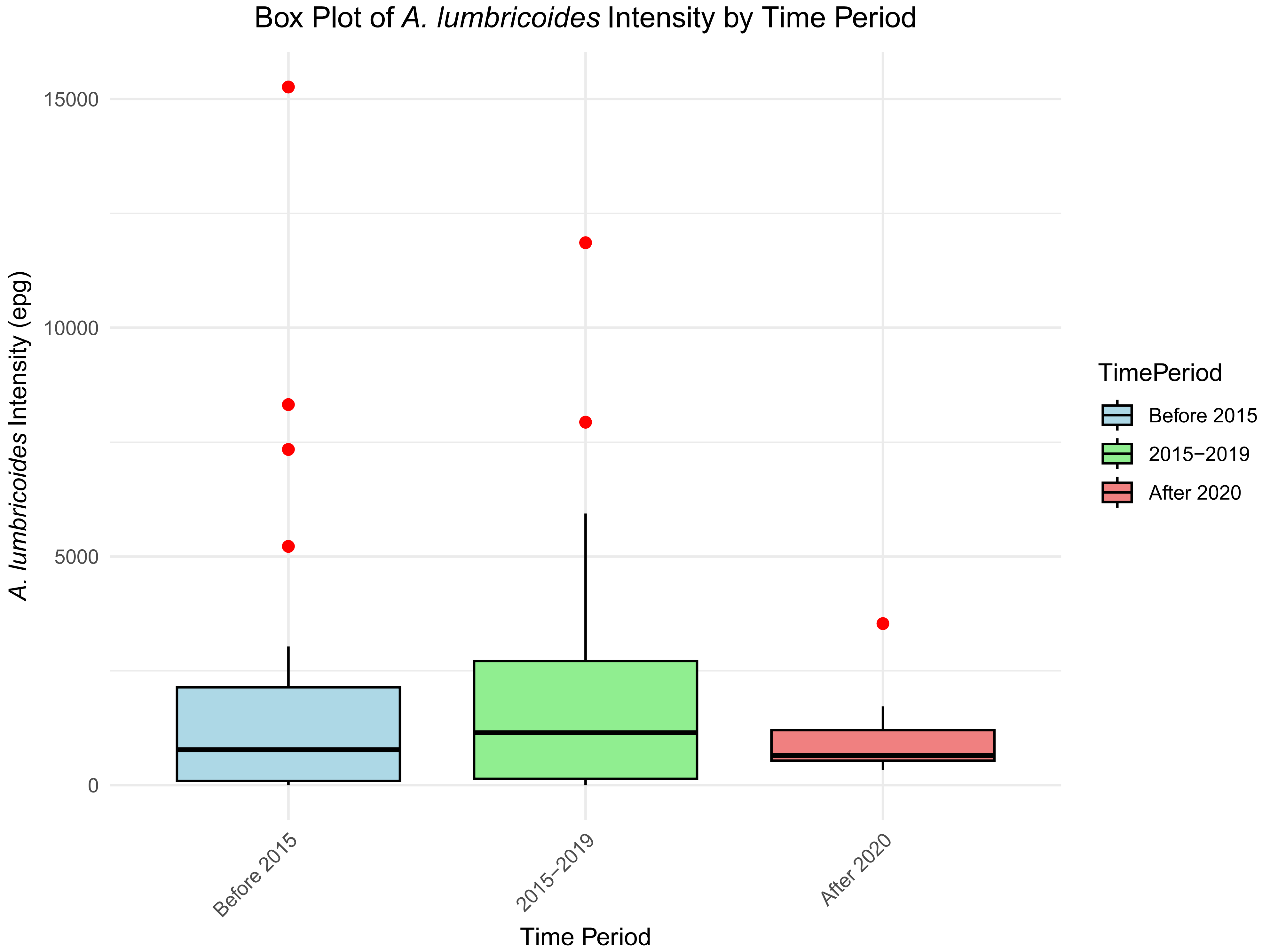

Additional file 2 Figure S1: Trends of *A. lumbricoides* mean egg count over the years.
