# Supplementary material for: Trends in the prevalence and intensity of soil-transmitted helminth (STH) infection in Ethiopia 2000 to 2023: a systematic review
Source: Parasit Vectors. 2025 Aug 9;18:340. doi: 10.1186/s13071-025-06928-3 (PMC12335801; doi:10.1186/s13071-025-06928-3)
Supplement: Supplementary file 3 — Additional file 3. Figure S2. Trends in T. trichiura mean egg count over the years [file 13071_2025_6928_MOESM3_ESM.pdf]

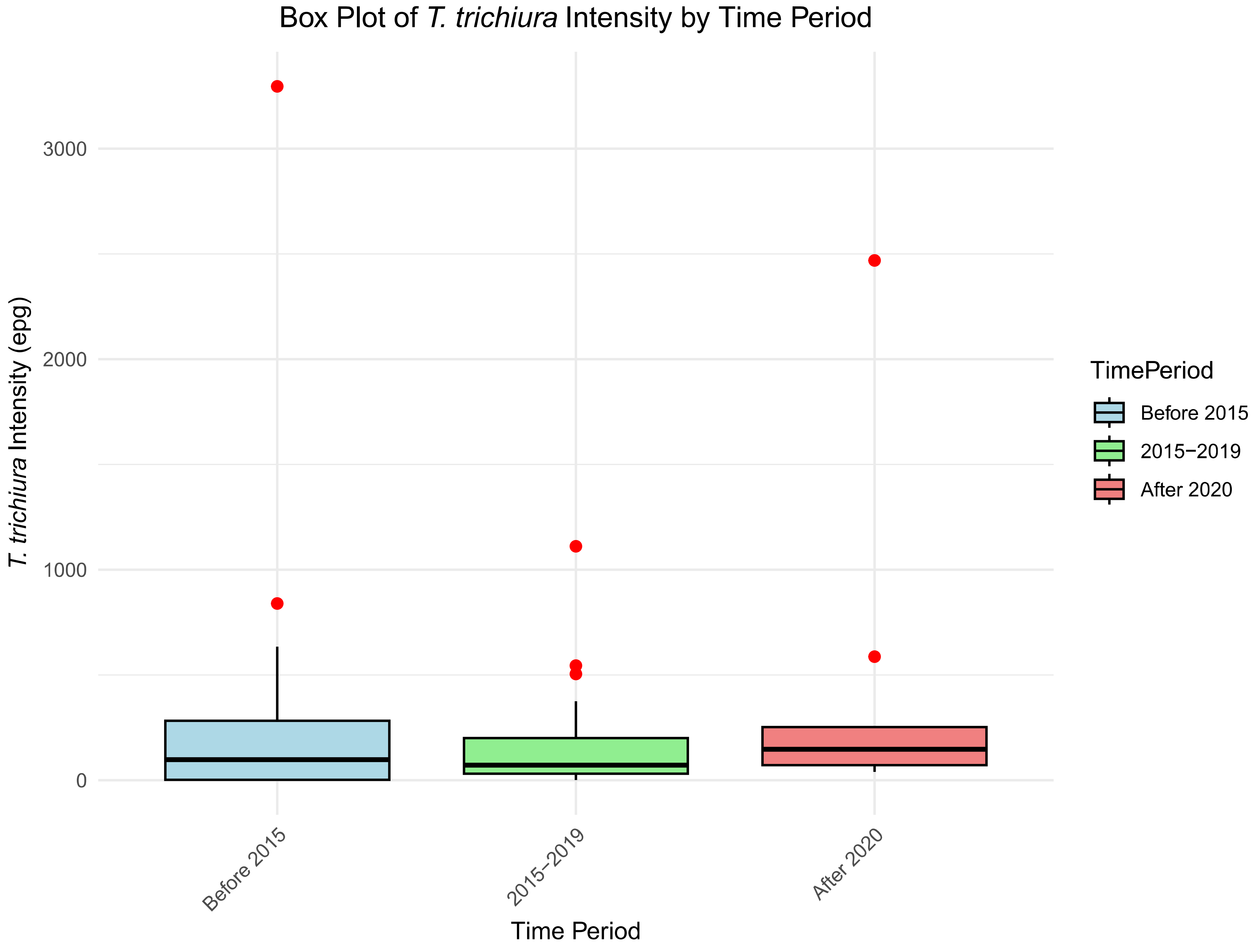

Additional file 3 Figure S2: Trends of *T. trichiura* mean egg count over the years.
